# Biophysical Properties of Somatic and Axonal Voltage-Gated Sodium Channels in Midbrain Dopaminergic Neurons

**DOI:** 10.3389/fncel.2019.00317

**Published:** 2019-07-10

**Authors:** Jun Yang, Yujie Xiao, Liang Li, Quansheng He, Min Li, Yousheng Shu

**Affiliations:** State Key Laboratory of Cognitive Neuroscience and Learning and IDG/McGovern Institute for Brain Research, Beijing Normal University, Beijing, China

**Keywords:** voltage-gated sodium (Nav) channels, action potential, sodium channel subtype, axon, dopaminergic neuron

## Abstract

Spiking activities of midbrain dopaminergic neurons are critical for key brain functions including motor control and affective behaviors. Voltage-gated Na^+^ channels determine neuronal excitability and action potential (AP) generation. Previous studies on dopaminergic neuron excitability mainly focused on Na^+^ channels at the somatodendritic compartments. Properties of axonal Na^+^ channels, however, remain largely unknown. Using patch-clamp recording from somatic nucleated patches and isolated axonal blebs from the axon initial segment (AIS) of dopaminergic neurons in mouse midbrain slices, we found that AIS channel density is approximately 4–9 fold higher than that at the soma. Similar voltage dependence of channel activation and inactivation was observed between somatic and axonal channels in both SNc and VTA cells, except that SNc somatic channels inactivate at more hyperpolarized membrane potentials (*V*_m_). In both SNc and VTA, axonal channels take longer time to inactivate at a subthreshold depolarization *V*_m_ level, but are faster to recover from inactivation than somatic channels. Moreover, we found that immunosignals of Nav1.2 accumulate at the AIS of dopaminergic neurons. In contrast, Nav1.1 and Nav1.6 immunosignals are not detectible. Together, our results reveal a high density of Na^+^ channels at the AIS and their molecular identity. In general, somatic and axonal channels of both SNc and VTA dopaminergic neurons share similar biophysical properties. The relatively delayed inactivation onset and faster recovery from inactivation of axonal Na^+^ channels may ensure AP initiation at high frequencies and faithful signal conduction along the axon.

## Introduction

Dopaminergic neurons in the midbrain play critical roles in many key brain functions including motor control, motivation, reward, and cognition (Nieoullon, 2002; Wise, 2004; Fields, 2007; Tobler et al., 2007). Dysfunction of dopaminergic systems will lead to major brain disorders, such as Parkinson’s disease, depression, schizophrenia, and addiction (Koob, 1998; Lewis and Lieberman, 2000; Dauer and Przedborski, 2003). There are two distinct groups of dopaminergic neurons located in substantia nigra pars compacta (SNc) and the ventral tegmental area (VTA). SNc neurons are essential circuit elements for controlling movement and learning new motor skills, while VTA neurons play a central role in emotive and motivational brain functions. In comparison with those in VTA, dopaminergic neurons in SNc are more vulnerable to degeneration in Parkinson’s disease.

Although they locate at different midbrain regions, dopaminergic neurons share similar electrophysiological properties. They discharge action potentials (APs) with broad voltage waveform in a pacemaking manner at a low frequency of 1–5 Hz and are able to generate bursts of APs at higher frequencies (10–30 Hz) *in vivo* (Grace et al., 2007; Tsai et al., 2009; Cao et al., 2010) and *in vitro* (Grace and Bunney, 1984; Hounsgaard et al., 1992; Johnson et al., 1992; Neuhoff et al., 2002; Korotkova et al., 2003; Kuwahara et al., 2006; Margolis et al., 2006; Khaliq and Bean, 2010). It has been proposed that tonic firing may determine the background dopamine level, while phasic (burst) firing is responsible for a large amount of dopamine release in response to reward signals (Grace et al., 2007; Tsai et al., 2009; Bermudez and Schultz, 2010). Optogenetic manipulation of the spiking activity in dopaminergic neurons would produce substantial behavior changes (Chaudhury et al., 2013; Tye et al., 2013; Gunaydin et al., 2014). Therefore, it is of interest to know what determines the excitability (i.e., AP generation) of dopaminergic neurons. Previous studies revealed differential mechanisms for pacemaking in these neurons. Spontaneous firing in VTA neurons is determined by a background Na^+^ current (Khaliq and Bean, 2010), whereas that in SNc neurons is driven by a subthreshold Ca^2+^ current (Chan et al., 2007, 2009). These findings suggest that ionic mechanisms governing AP generation in dopaminergic neurons could be different.

Voltage-gated Na^+^ channels (Nav) are critical for AP generation in CNS neurons. Biophysical properties of Nav in dopaminergic neurons differ from those of midbrain GABAergic neurons (Seutin and Engel, 2010; Ding et al., 2011). The predominant pore-forming α subunits in the brain are Nav1.1, Nav1.2, and Nav1.6. Subunit Nav1.3 is also expressed but mainly during earlier development (Shah et al., 2001). Different channel subunits may have distinct biophysical properties. For example, Nav1.6 activates at much lower membrane potential (*V*_m_) levels than Nav1.2 (Rush et al., 2005). In neocortical pyramidal cells, Nav1.6 accumulates at distal regions of the axon initial segment (AIS) and contributes to AP initiation, whereas Nav1.2 concentrates at peri-somatic regions and promotes AP backpropagation to the soma and dendrites (Hu et al., 2009). Previous findings from single-cell RT-PCR suggest expression of Nav1.1, Nav1.2, and Nav1.6 in substantia nigral dopaminergic neurons (Ding et al., 2011). It remains unknown whether midbrain dopaminergic neurons express these channel subunits at the protein level and whether there is any difference in the biophysical properties between SNc and VTA cells. Since AIS is the AP initiation site, it is of interest to examine whether AIS Nav channels differ from those at the soma.

To address these questions, we performed electrophysiological recording and channel subunit antibody staining experiments. We found that, in general, Na^+^ channels of dopaminergic neurons share similar biophysical properties in both SNc and VTA. In comparison with those at soma, axonal channels take longer time to inactivate at a depolarizing *V*_m_ level but show faster recovery from inactivation. As compared to those at the soma, AIS channels have a much higher density. These features ensure AP initiation at high frequencies at the AIS and faithful conduction. In contrast, properties of somatic channels may help gate AP backpropagation to the somatodendritic compartments. Interestingly, different from previous findings, our immunostaining experiments reveal a high-level expression of Nav1.2 at the AIS of both SNc and VTA dopaminergic neurons, while Nav1.6 and Nav1.1 immunosignals are undetectable.

## Results

### AP Initiation and Propagation in Midbrain Dopaminergic Neurons

**FIGURE 1:**
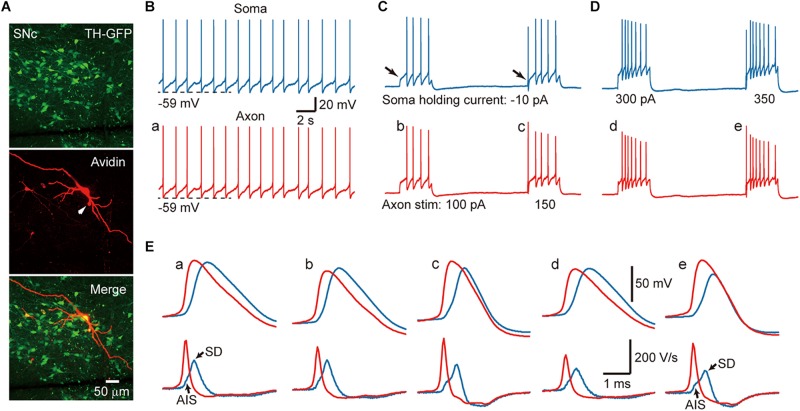
Action potentials (APs) are initiated at the AIS of SNc TH-positive neurons. **(A)** An example recorded SNc TH-GFP neuron. **(B)** Dual whole-cell recording from the soma (blue) and the axon (red, 10 μm away from the soma) showing pace making activity at both recording sites. **(C)** Negative DC current injection to the soma prevents spontaneous firing. Positive current pulses (500 ms in duration) at the AIS cause repetitive firing at the axon and the soma. Note the absence and presence of the initial AP (arrows) in response to weak and strong current pulses (100 vs. 150 pA). **(D)** Injection of current pulses to the soma evoke repetitive firing at both recording sites. Also note the absence of presence of the initial AP. **(E)** Top, expansion of the APs indicated in **B–D**. Axonal APs precede the somatic APs in all cases. Bottom, the first derivative (d*V*/dt) of the corresponding APs. The two components at the rising phase of the somatic d*V*/dt trajectory (blue) correspond to the AIS and SD potentials, respectively. Note the difference of the rising phase between somatic and axonal d*V*/dt traces.

In acute midbrain slices from TH-GFP mice maintained at room temperature (24–26°C), we performed whole-cell recordings from the soma and/or the axon of GFP-positive cells. Most of the recorded neurons emitted axons from one of their proximal thick dendrites (Figure 1A), known as the axon-bearing dendrite (ABD). Axon recordings were obtained from the blebs formed at AIS, less than 120 μm away from the ABD branching site. In current-clamp mode, 56% of the recorded SNc cells (*n* = 10/18) showed spontaneous firing with an average frequency of 1.5 ± 0.6 Hz, 43% of VTA cells discharged spontaneously (*n* = 6/14) at a frequency of 1.35 ± 1.2 Hz. As shown in Figure 1B, the spontaneous APs of a SNc cell could be detected at both the somatic and axonal recording sites (*n* = 10 cells). Hyperpolarizing the *V*_m_ by injecting a small constant negative current (−10 to −90 pA) to the soma would prevent spontaneous firing. Delivery of 500-ms positive current pulses to the axon (Figure 1C) or the soma (Figure 1D) could evoke trains of APs at both recording sites.

Close examination of the AP waveforms revealed that the AIS APs preceded the somatic APs no matter whether they are spontaneous and where the current pulses were applied (Figure 1E), indicating that AP initiated first at the axon. Previous studies (McCormick et al., 2007; Hu et al., 2009) indicated that, if APs initiate near the recording site, the *V*_m_ will show smooth transition to the AP upstroke because of accumulative activation of voltage-gated Na^+^ channels; however, if APs initiate at a remote site, the *V*_m_ will first depolarize due to AP propagation and then generate a rapid upstroke due to the recruitment of local Na^+^ channels. Indeed, the first derivative (d*V*/dt) of individual somatic APs showed two components in the AP rising phase, corresponding to the arrival of AIS potential and the generation of somatodendritic (SD) potential, respectively (blue, Figure 1E). In sharp contrast, there was no bump at the rising phase of AIS APs (red, Figure 1E). These observations agree well with an initiation zone at the AIS (McCormick et al., 2007).

Previous studies in other types of neurons showed that the voltage-gated Na^+^ channels at the AIS possess higher density (Kole et al., 2008; Hu et al., 2009, 2014; Li et al., 2014) and unique biophysical properties (Hu et al., 2014), which are critical for AP initiation at the AIS. We, therefore, sought to investigate differences in the density and the voltage dependence between somatic and axonal Na^+^ channels.

### Density of Somatic and AIS Na^+^ Channels

**FIGURE 2:**
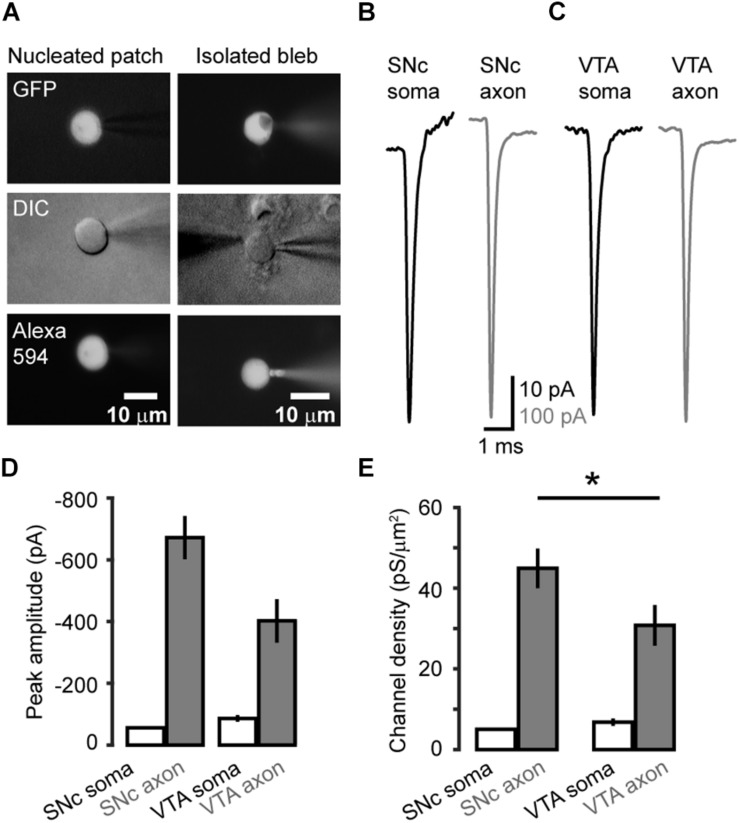
Na^+^ channel density at the soma and the AIS of midbrain TH neurons. **(A)** Fluorescence and DIC images of the recorded somatic nucleated patch and isolated AIS bleb. The recording pipettes contained Alexa Fluo-594, which could label the recorded nucleated patch and axonal bleb. **(B,C)** Example Na^+^ currents obtained from SNc **(B)** and VTA cells **(C)** by stepping the holding voltage from −120 mV (35 ms) to 0 mV (25 ms). **(D)** Averaged peak amplitudes of Na^+^ currents. **(E)** Averaged channel density at the soma and the AIS of SNc and VTA cells. ^*^p < 0.05, comparison between SNc and VTA axonal channel density.

We carried out voltage-clamp recordings from somatic nucleated patches and isolated AIS blebs of GFP-positive neurons in SNc and VTA (Figure 2A). Transient inward Na^+^ currents were evoked by stepping the *V*_m_ from a 35-ms prepulse at −120 mV to a series of testing pulses (25 ms in duration) from −70 to +25 mV. The maximal peak amplitudes of the evoked currents in isolated axonal blebs of SNc (*n* = 22, Figure 2B) and VTA cells (*n* = 22, Figure 2C) were 672 ± 70 and 402 ± 70 pA (Mean ± SEM, Figures 2B–D), and the calculated conductance densities were 44.9 ± 4.9 and 30.8 ± 5.0 pS/μm^2^, respectively (Figure 2E). In contrast, currents obtained from somatic nucleated patches were much smaller (Figures 2A–C). The maximal current amplitudes of SNc (*n* = 14) and VTA cells (*n* = 10) were 56.0 ± 4.0 and 86.1 ± 10.7 pA, corresponding to conductance densities of 5.0 ± 0.3 and 6.8 ± 0.9 pS/μm^2^, respectively (Figures 2D,E), which were approximately 9 and 4.5 fold lower than those in SNc (*p* = 1.2 × 10^–6^) and VTA AIS (*p* = 1.9 × 10^–4^).

Together, these results reveal a high density of Na^+^ channels at the AIS in both SNc and VTA cells. Interestingly, the channel density in SNc AIS is significantly higher than that of VTA AIS (*p* = 0.03), whereas the density of somatic channels in SNc is similar to that of VTA (*p* = 0.15).

### Voltage-Dependent Activation and Deactivation

**FIGURE 3:**
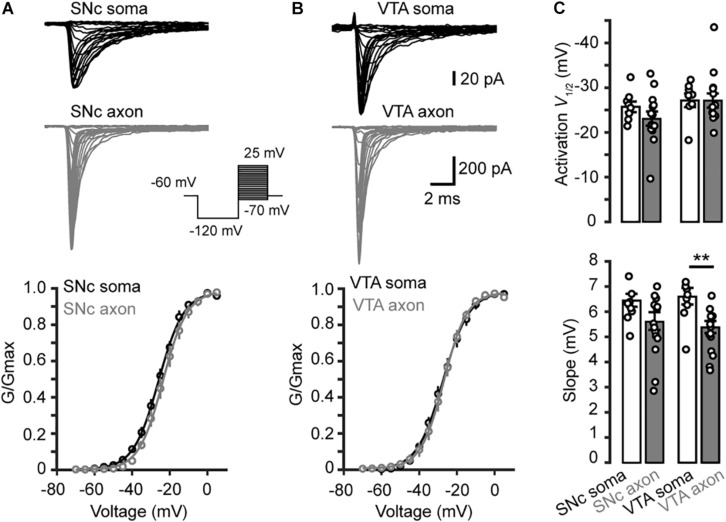
Activation of transient Na^+^ currents in somatic nucleated patches and isolated AIS blebs. **(A)** Top, Na^+^ currents evoked by activation voltage commands (inset) in a somatic nucleated patch and an isolated axonal bleb in SNc. Bottom, activation curves from somatic and axonal Na^+^ currents of SNc cells. The plots are fitted with Boltzmann functions. **(B)** Similar as in **A** but from VTA cells. **(C)** Group data showing the half-activation voltage (*V*_1/2_) and the slope of Na^+^ channel activation curves. ^∗∗^*p* < 0.01.

Examination of the Na^+^ currents evoked by the activation voltage steps (Figures 3A,B) revealed similar voltage dependence between somatic and axonal Na^+^ channels in both SNc and VTA cells (Figure 3C). Currents started to activate around −50 mV and reached complete activation around 0 mV. Fitting the activation curves with Boltzmann equation yielded the half-activation voltages (*V*_1/2_) and slopes (*k*). The average *V*_1/2_ for somatic and axonal channels of SNc cells were −25.7 ± 1.2 (*n* = 8) and −23.0 ± 1.5 mV (*n* = 14, *p* = 0.18), respectively; the slopes were 6.4 ± 0.2 and 5.6 ± 0.3 mV (*p* = 0.14, Figure 3C). Similar half-activation voltages were obtained from VTA cells (−27.1 ± 1.5 for soma and −25.7 ± 1.0 mV for axon, *p* = 0.23); the slopes, however, showed a slight but significant difference (6.6 ± 0.3 vs. 5.4 ± 0.2 mV, *p* = 0.004; *n* = 8 for soma and 13 for axon, Figure 3C).

**FIGURE 4:**
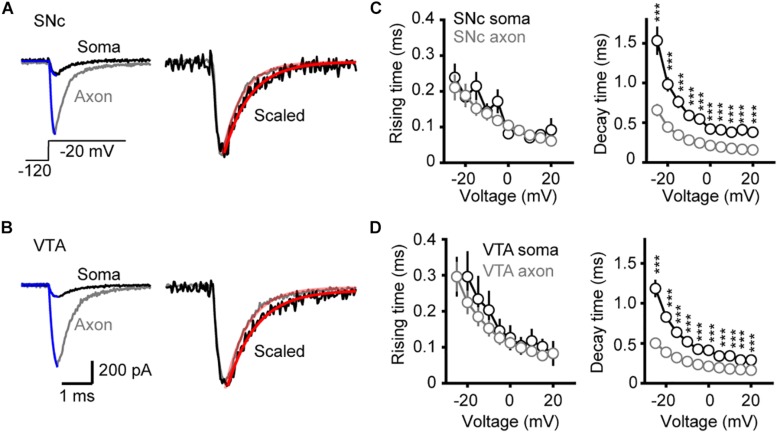
Kinetics of activation and inactivation of Na^+^ currents. **(A,B)** Example Na^+^ currents of SNc **(A)** and VTA cells **(B)** evoked by stepping the voltage from a 35-ms prepulse of –120 to a test pulse of –20 mV. The rising phase of the currents was fitted by an exponential function with a delayed onset. The decay phase was fitted with a single exponential function. **(C,D)** Comparison of the rise and decay time between somatic and axonal Na^+^ currents. ^∗∗∗^*p* < 0.001.

To examine the kinetics of channel activation and inactivation, we fitted the rising and the falling phases of transient currents with single exponential functions (Figures 4A,B). We observed no significant difference in the rise time between somatic and axonal currents evoked by steps to a range of voltage levels (−25 to +20 mV) in both SNc and VTA cells (SNc: *n* = 8 nucleated patches and 14 isolated blebs; VTA: *n* = 8 and 13, respectively, Figures 4C,D). However, the decay time constants of axonal currents were significantly smaller than those of somatic currents for all the tested voltage levels (Figures 4C,D), indicating that axonal channels inactivate faster than somatic ones. These results indicate that, although somatic and axonal Na^+^ channels share similar voltage dependence, they do differ in some aspects including the channel inactivation kinetics.

**FIGURE 5:**
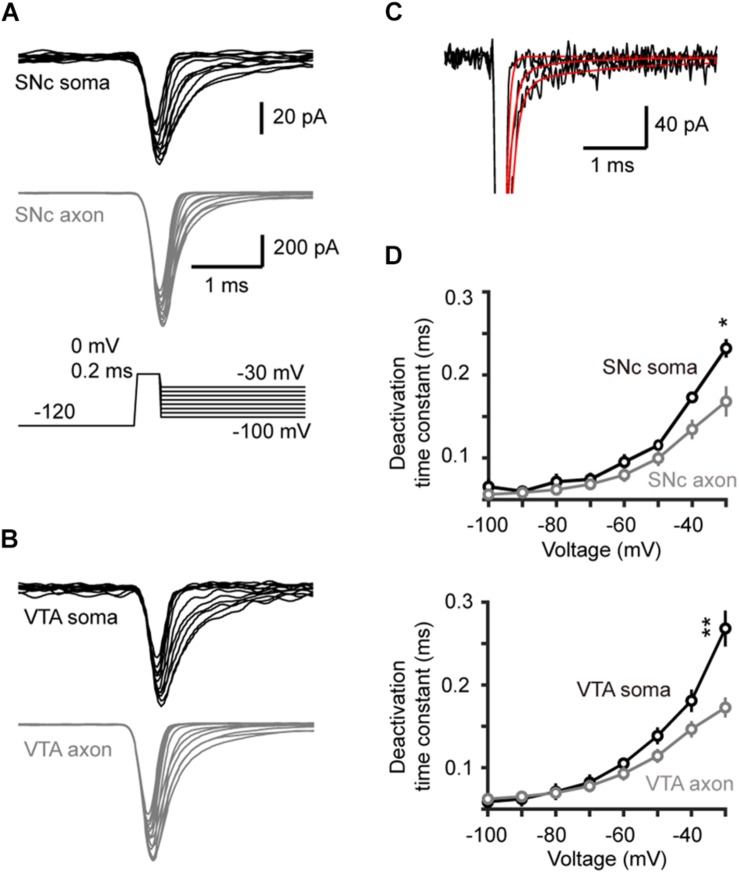
Time course of Na^+^ channel deactivation in TH neurons. **(A,B)** Families of Na^+^ currents in SNc **(A)** and VTA cells **(B)** evoked by the channel deactivation voltage protocol (inset). Immediately after the test pulse (from a 30-ms –120 mV prepulse to 0 mV, 0.2 ms), the Vm was held at different levels (from –100 to –30 mV with increment of 10 mV). **(C)** Fitting the decay of the currents with single exponential functions. **(D)** The deactivation time constants at different voltage levels in SNc (top) and VTA cells (bottom). ^*^p < 0.05; ^∗∗^*p* < 0.01.

We next measured the time constant of channel deactivation. Na^+^ channels were activated by stepping the *V*_m_ from −120 to a 0.2-ms test pulse (0 mV), which was followed by a series of voltage steps (from −100 to −30 mV, Figures 5A,B). We fitted the tail currents with single exponential functions to obtain the time constants at different *V*_m_ levels (Figure 5C) and found that axonal channels deactivate more rapidly than somatic channels. The time constant of axonal channels at −30 mV was significantly shorter than that of somatic channels in SNc cells (0.17 ± 0.02 vs. 0.23 ± 0.01 ms, *n* = 5 nucleated patches and 14 isolated blebs, *p* = 0.04, Figure 5D). Similar results were observed in VTA cells (soma: 0.27 ± 0.01 ms, *n* = 5; AIS: 0.17 ± 0.01 ms, *n* = 14, *p* = 0.002, Figure 5D).

### Steady-State Inactivation and Recovery

**FIGURE 6:**
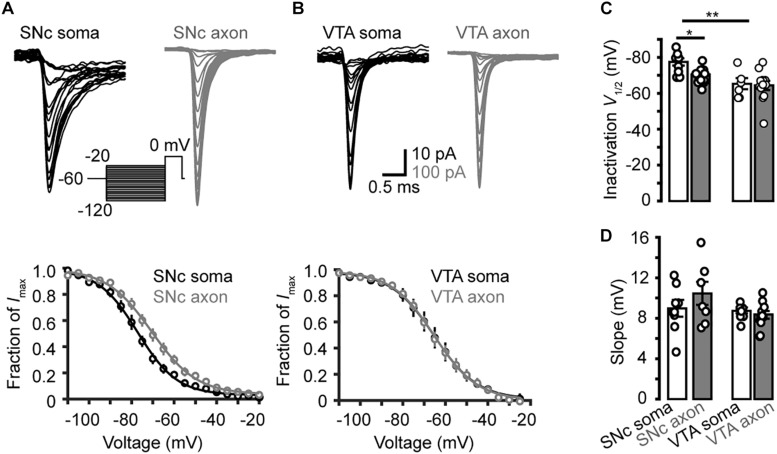
Steady-state inactivation of transient Na^+^ currents in midbrain TH neurons. **(A)** Example families of Na^+^ currents (top) evoked by the voltage commands (inset) for steady-state channel inactivation in a SNc somatic nucleated patch and an isolated AIS bleb. Bottom, inactivation curves of SNc Na^+^ currents (*n* = 7 somatic nucleated patches and 7 isolated AIS blebs). **(B)** Similar as in A but from VTA cells. **(C,D)** Group data comparing the half-inactivation voltages and the inactivation curve slopes. ^*^p < 0.05; ^∗∗^*p* < 0.01.

Next, we examined the steady-state inactivation (channel availability) at different *V*_m_ levels. Transient Na^+^ currents were evoked by stepping the *V*_m_ from a series of conditioning pulses (from −120 to −20 mV, 35 ms) to the 20-ms test pulse at 0 mV (Figures 6A,B). The half-inactivation voltage of SNc somatic channels were −77.5 ± 2.0 mV (*n* = 7), significantly lower than that of axonal channels (−70.4 ± 2.0 mV, *p* = 0.03); no significant difference in the slope was observed (9.0 ± 0.8 mV for soma vs. 10.4 ± 1.2 mV for axon, *p* = 0.4, *n* = 8, Figures 6A,C,D). For VTA cells, the *V*_1/2_ of inactivation and slope of somatic channels were −65.3 ± 3.1 mV and 8.7 ± 0.3 mV (*n* = 6), and those of axonal channels were −64.4 ± 2.5 mV (*p* = 0.9) and 8.4 ± 0.4 mV (*n* = 12, *p* = 0.4, Figures 6B,D), respectively. As shown in Figure 6C, the half-inactivation voltage of SNc somatic channels was also significantly hyperpolarized than that of VTA somatic ones (*p* = 0.01). These results suggest that, at subthreshold *V*_m_ levels (from −80 to −55 mV), somatic channels of SNc cells show much lower availability for AP generation than their axonal channels; in addition, they are also less available than channels at both the soma and the axon of VTA cells.

**FIGURE 7:**
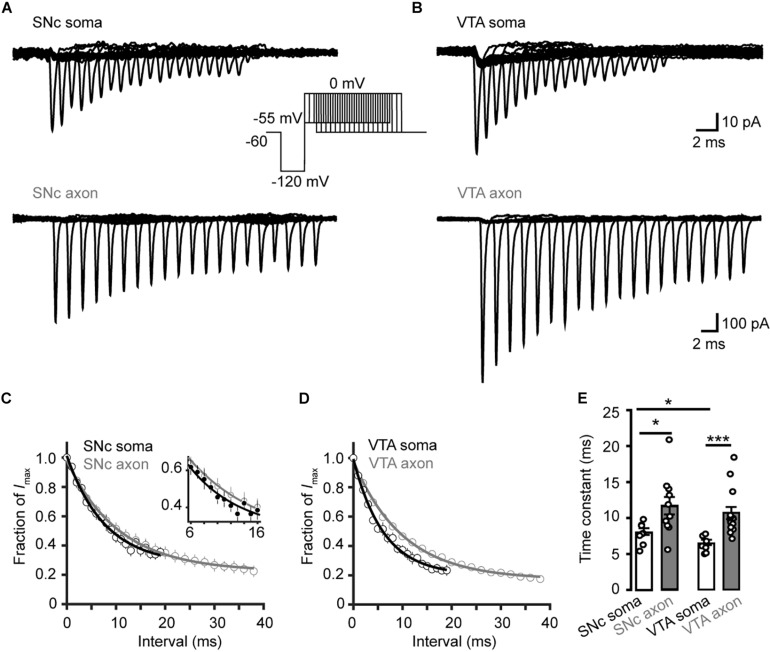
Time course of Na^+^ channel inactivation onset in TH neurons. **(A)** Example currents evoked by the voltage commands (inset) in SNc neurons. The *V*_m_ was stepped from a prepulse of –120 mV (20 ms) to –55 mV with varying intervals followed by a test pulse to 0 mV (5 ms). **(B)** Similar as in A but from VTA neurons. **(C,D)** Plots of the currents as a function of intervals. Currents were normalized to the Na^+^ currents without time interval. The plots were fitted with single exponential functions. Inset, expanded plots showing differences between the two groups. **(E)** Time constants for the development of Na^+^ channel inactivation at –55 mV. ^*^p < 0.05; ^∗∗∗^*p* < 0.001.

To investigate the time course of the onset of channel inactivation, we applied a prepulse to −120 mV for 15 ms and then stepped the *V*_m_ to −55 mV, followed by a test pulse to 0 mV after a delay ranging from 0 to 38 ms (Figures 7A,B). Single exponential fits of the normalized currents yielded time constants of 8.04 ± 0.52 and 6.40 ± 0.41 ms for SNc (*n* = 8 cells) and VTA somatic channels (*n* = 7 cells), respectively, significantly faster than those of axonal channels (SNc: 11.7 ± 1.2 ms, *n* = 11, *p* = 0.02; VTA: 10.6 ± 0.8 ms, *n* = 15, *p* = 4.0 × 10^–4^, Figures 7C–E).

**FIGURE 8:**
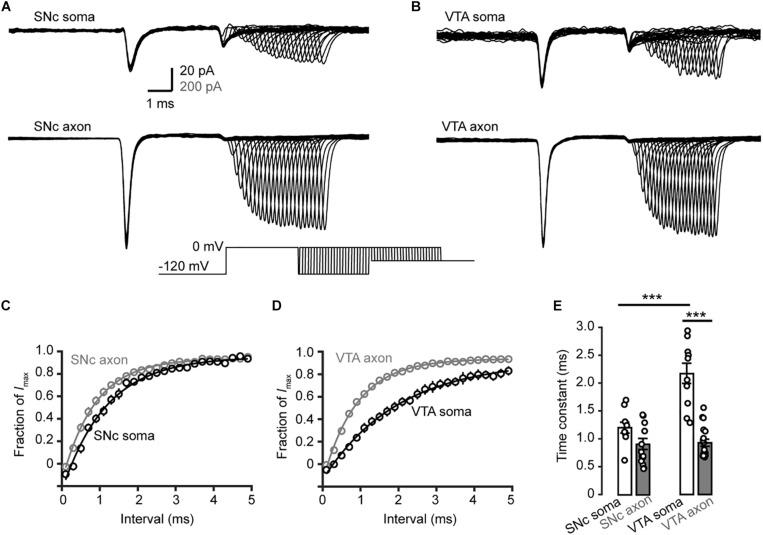
Recovery from inactivation of Na^+^ channels in TH neurons. **(A,B)** Representative currents evoked by two paired pulses (from –120 to 0 mV) with varying intervals (inset) in SNc **(A)** and VTA neurons **(B)**. **(C,D)** Plots of the currents in SNc **(C)** and VTA cells **(D)** as a function of intervals between the two pulses. Currents were normalized to the peak amplitude of the current evoked by the first pulse. **(E)** Comparison of the recovery time constants from inactivation between Na^+^ currents in different groups. ^∗∗∗^*p* < 0.001.

We then examined the time course of recovery from channel inactivation. After the prepulse (−120 mV), paired pulses with different intervals (from 1 to 110 ms at −120 mV) were applied to the patches (Figure 8). Single exponential fits revealed a tendency of axonal Nav channels to be faster to recover from inactivation than somatic channels (soma: 1.2 ± 0.1 ms, *n* = 10, vs. axon: 0.9 ± 0.1 ms, *n* = 14, *p* = 0.11, Figures 8A,C). Similarly, somatic channels of VTA cells showed a slower recovery from inactivation than axonal channels (soma: 2.2 ± 0.2 ms vs. axon: 0.9 ± 0.1 ms, *n* = 10 nucleated patches and 19 isolated blebs, *p* = 1.0 × 10^–5^, Figures 8B,D,E) as well as SNc somatic channels (Figure 8E). These results suggest that axonal channels recover from inactivation faster than somatic channels, ensuring high-frequency AP generation and conduction.

### Molecular Identity of Axonal Na^+^ Channels

**FIGURE 9:**
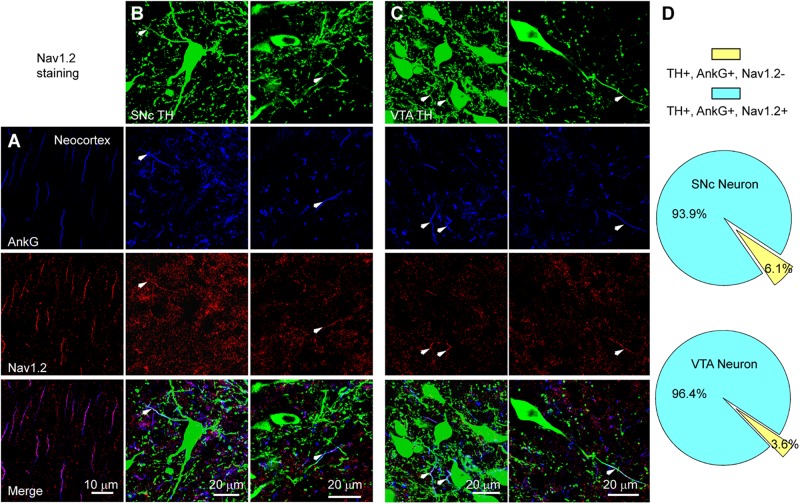
The AIS of midbrain TH-positive neurons express Nav1.2. **(A)** Double staining of AnkG (Blue) and Nav1.2 (Red) in neocortex. **(B)** Triple staining of TH (green), AnkG (Blue) and Nav1.2 (Red) in two example SNc cells. White arrowheads indicate neurite segments positive to all three antibodies. **(C)** Similar as in B but for cells in VTA. **(D)** Percentages of TH/AnkG-positive neurites in SNc (top) and VTA (bottom) that are positive (blue) or negative (yellow) to Nav1.2 staining.

**FIGURE 10:**
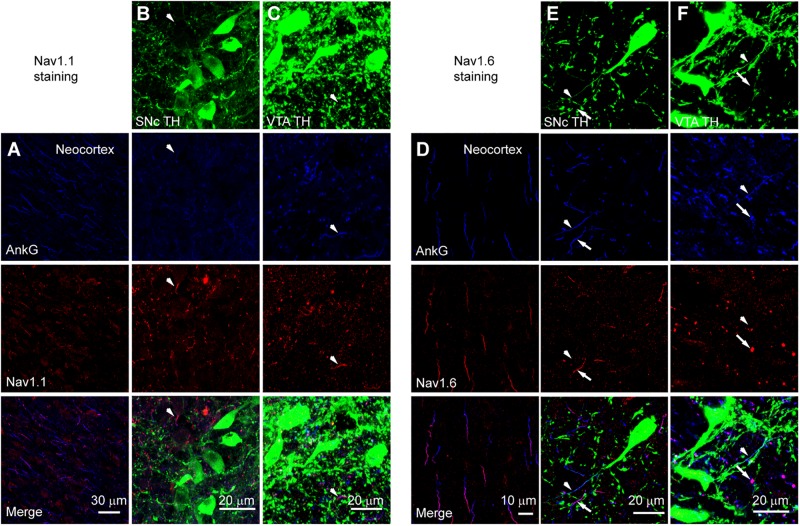
Absence of Nav1.1 and Nav1.6 from the AIS of midbrain TH-positive neurons. **(A)** Double staining of AnkG (Blue) and Nav1.1 (Red) in neocortex. **(B)** Triple staining of TH (green), AnkG (Blue) and Nav1.1 (Red) in SNc. White arrowheads indicate neurites positive to AnkG and Nav1.1 but negative to TH. **(C)** Similar as in B but for VTA. **(D–F)** Similar to **A–C** but for Nav1.6 staining in the neocortex **(D)**, SNc **(E)** and VTA **(F)**. Note the absence of Nav1.6 immunosignals from the TH-positive neurites (arrowheads). Arrows indicate neurites positive to AnkG and Nav1.6 but negative to TH.

We performed triple immunostaining using antibodies of TH, Ankyrin G (AnkG), and different Na^+^ channel α subunits, including Nav1.2, Nav1.1, and Nav1.6. TH and AnkG were used to identify the dopaminergic neurites and AIS, respectively. We performed Nav1.2 antibody staining in the neocortex as a positive control (Hu et al., 2009; Li et al., 2014; Figure 9A). In both SNc and VTA (Figures 9B,C), most of the neurites positive to TH and AnkG were also positive to Nav1.2. Immunosignals of Nav1.2 were found in 93.9% and 96.4% of the neurites positive to both TH and AnkG (*n* = 46 in SNc and 54 in VTA from 6 mice, Figure 9D). However, none of the TH/AnkG-positive neurites in both SNc and VTA showed Nav1.1 signals (*n* = 50 from 3 mice, Figures 10A–C), although positive signals were found in the neocortex (Figure 10A) and in some of the neurites positive to AnkG but negative to TH (Figures 10B,C). These results indicate that the AIS of dopaminergic neurons express the α subunits Nav1.2 but not Nav1.1. Similar to that in the neocortex (Li et al., 2014) the AIS of TH-negative GABAergic neurons within SNc and VTA may express the subtype Nav1.1. Surprisingly, the most abundant α subunit Nav1.6 in the brain was not found in the AIS of TH-positive neurons (*n* = 50 from 3 mice, Figures 10D–F). Similar to the pattern of Nav1.1, Nav1.6 signals were found in TH-negative but AnkG-positive neurites, suggesting an expression of this channel subtype in other types of neurons in the midbrain. We found no detectible signals at the soma using channel antibodies, possibly due to the low abundance of channels. The molecular identity of somatic channels remains to be further examined. Together, these results indicate that Nav1.2 is the predominant α subunit in the AIS of midbrain dopaminergic neurons.

## Discussion

In this study, we reveal a high density of voltage-gated Na^+^ channels at the AIS of putative midbrain dopaminergic neurons, which may determine the AIS as the AP initiation zone. Although the activation of voltage-gated Na^+^ channels at the soma and the AIS in midbrain dopaminergic neurons share similar voltage dependence, channel inactivation differs in many aspects. First, the inactivation of transient Na^+^ currents at the axon is much faster than that of somatic channels. Second, at a subthreshold depolarizing *V*_m_ level, inactivation onset of axonal channels is more delayed than that of somatic channels. Finally, axonal channels show faster recovery from inactivation. These inactivation properties allow higher channel availability, faster kinetics, and more rapid recovery from inactivation, to ensure the generation of APs at higher frequencies and depolarized *V*_m_ levels. Interestingly, our immunostaining experiments indicate that Nav1.2 is the predominant channel subtype at the AIS that governs AP initiation.

### AP Initiation and Channel Density at the AIS

Similar to other types of neurons in the brain including neocortical pyramidal cells (Stuart et al., 1997; Chan et al., 2009) and cerebellar Purkinje cells (Clark et al., 2005), the AP initiation site of midbrain dopaminergic neurons locates at the AIS. Our dual whole-cell recordings at the soma and the AIS in both SNc and VTA neurons indicate that AIS APs always precede the somatic APs no matter whether the APs are generated spontaneously or evoked by current pulses (Figure 1). The phase plots of somatic APs have two components during the upstrokes, corresponding to the AIS potential and SD potential, respectively. However, the upstrokes of AIS APs show smooth initial depolarization and absence of the double components (McCormick et al., 2007), indicating that APs are initiated at the AIS. In agreement with this, simultaneous recordings from the soma and dendrites of dopaminergic neuron show that APs in ABD occur earlier than those at the soma and non-ABD, suggesting that APs originate at the axon (Hausser et al., 1995; Gentet and Williams, 2007).

Previous studies suggest roles of specific channel subtypes and high channel density in determining the AP initiation site (Stuart et al., 1997; Li et al., 2014). The Na^+^ channel density at the AIS is about ∼34 fold higher than that at the soma in neocortical pyramidal cells (Hu et al., 2009). The AIS channel density is also higher in neocortical (∼60 fold) (Hu et al., 2009) and hippocampal parvalbumin-expressing neurons (Hu et al., 2009) ensuring fast signal conduction along the axon. In comparison with these types of cells, dopaminergic neurons express relatively less Na^+^ channels at their AIS, the channel density is only 4–9 fold that of somatic ones. Distinct from our estimation of channel density at the soma, previous studies revealed a higher density in rat midbrain cells (Seutin and Engel, 2010; Moubarak et al., 2019). The discrepancy could be attributable to the identification of putative dopaminergic neuron, recording configuration, and animal species. Since dopaminergic neurons are spontaneously active, changes in mean frequency of their tonic firing may control and regulate the background dopamine level in the target brain regions (Sulzer et al., 2016), leading to relatively slow neuromodulation and alteration of brain states. Therefore, precision of individual APs could be not essential for dopaminergic neuron signaling. Activation of AIS channels not only initiates APs but also provides currents to charge the somatodendritic membrane and determine whether or not an AP can successfully backpropagate to the soma and dendrites. Considering that AP backpropagation play critical roles in mediating somatodendritic dopamine release (Geffen et al., 1976; Rice and Patel, 2015) and is subject to modulation by D2 receptors (Cragg and Greenfield, 1997; Gentet and Williams, 2007), we speculate that the lower channel density at the AIS of dopaminergic neurons, as compared with that of pyramidal cells, may ensure a higher sensitivity of AP backpropagation to modulation by dopamine and other neuromodulators.

### Voltage Dependence of Channel Activation and Inactivation

We found surprisingly that somatic and AIS Na^+^ channels share similar voltage dependence of channel activation in both SNc and VTA cells (Figure 3). The half-activation voltage shows no significant difference (ranging from −23 to −27 mV). Activation curves of somatic and axonal channels differ slightly in their slopes; small but significant difference was only found in VTA cells (soma: 6.6 mV vs. AIS: 5.4 mV). In addition, activation kinetics of somatic channels are similar to those of AIS channels, as reflected by their similar rising time (Figure 4). The voltage dependence of channel inactivation, however, is more complicated. In the experiments examining the steady-state channel inactivation (availability), we found that SNc somatic channels have the most hyperpolarized half-inactivation voltage than AIS channels in SNc and both somatic and AIS channels in VTA, indicating that at similar *V*_m_ levels SNc soma has the lowest channel availability (Figure 6). Different from SNc, VTA somatic channels share similar voltage dependence with AIS channels. In general, AIS channels of both SNc and VTA cells show slower onset of inactivation and faster recovery from inactivation as compared with somatic channels (Figures 7, 8).

The difference in the inactivation curves may reflect differences in channel subunits or modulation of channel proteins. Since the expression level of Na^+^ channels at the soma is extremely low and the antibody staining is not sensitive enough, the molecular identity of somatic channels remains unclear. However, our immunostaining results (Figures 9, 10) together with findings from previous *in situ* hybridization experiments (Gonzalez-Cabrera et al., 2017) indicate that Nav1.2 subunits preferentially express in dopaminergic neurons. In contrast, Nav1.1 and Nav1.6 may only express in other types of neurons, possibly GABAergic inhibitory neurons. Therefore, it is unlikely that the channel subunit difference is attributable to the difference in the inactivation curves of SNc somatic and axonal channels. In comparison to Nav1.6 channels that are refractory to modulation by PKA or PKC (Maurice et al., 2001; Chen et al., 2009), Nav1.2 channels are much more vulnerable to modulation by neuromodulators and protein kinases (Maurice et al., 2001). PKC causes phosphorylation of serine 1506 at the inactivation gate (West et al., 1991) while PKA induces phosphorylation in the I–II loop but not the III–IV loop that is responsible for channel inactivation (Cantrell and Catterall, 2001). Phosphorylation of Nav1.2 induced by PKA and PKC would increase the slow channel inactivation (Carr et al., 2003; Chen et al., 2006, 2008). Therefore, we speculate that the differences in channel inactivation curves as well as those of inactivation and deactivation time courses could be due to differential phosphorylation of somatic and axonal channels. However, it remains unknown what determines the phosphorylation level and whether somatodendritic release of dopamine plays a role in the differential regulation.

Previous studies in neocortical pyramidal cells and inhibitory interneurons reveal distinct voltage-dependent properties of Na^+^ channels in the soma and the axon (Hu et al., 2009, 2014; Li et al., 2014; Ye et al., 2018). The half-activation voltages of channels distributed in distal AIS regions are more hyperpolarized than those in the soma. In midbrain dopaminergic neurons, the similarities in voltage dependence of channel activation at the soma and AIS suggest a more important role of the high channel density and the thin AIS structure in AP initiation (Stuart et al., 1997). In comparison with that of AIS channels of pyramidal cells, the relatively depolarized half-activation voltage of AIS channels would increase the AP threshold and slow down the conduction velocity along the axon. The slower development of channel inactivation and faster recovery ensure higher channel availability in prolonged depolarization levels and with a high firing rate, such as during phasic firing. It remains to be further examined whether biophysical properties of somatic and axonal channels are subject to modulation by neuromodulator receptors.

### Molecular Identity of AIS Na^+^ Channels

Previous findings (Ding et al., 2011) from single-cell RT-PCR suggest that dopaminergic neurons express all four Na^+^ channel subtypes in the central nervous system, including Nav1.1, Nav1.2, Nav1.3, and Nav1.6. In addition, quantitative RT-PCR reveal relatively higher expression levels of Nav1.2 in dopaminergic neurons but Nav1.1 and Nav1.6 in GABAergic neurons of the substantial nigra. *In situ* hybridization experiments also revealed that most of the SNc neurons express mRNA of Nav1.2, only a small population contains mRNA of Nav1.6 (Gonzalez-Cabrera et al., 2017). In agreement with these findings, our immunostaining results reveal that, at the protein level, Nav1.2 is the predominant channel subtype at the AIS of TH-expressing neurons in both SNc and VTA (Figure 9). In sharp contrast, Nav1.1 and Nav1.6 immunosignals are not detectible in TH-positive cells (Figure 10). Immunosignals of Nav1.1 and Nav1.6 are found in some TH-negative neurites, most likely from local GABAergic neurons. Considering that Nav1.3 is mainly expressed during earlier developmental stages (Shah et al., 2001) and has a much lower expression level in juvenile animals as compared with other subunits in single-cell RT-PCR experiments (Ding et al., 2011), we did not examine the expression of this channel subtype in the immunostaining experiments.

The exclusive expression of Nav1.2 at the AIS of dopaminergic neurons suggest a critical role of Nav1.2 in determining the excitability of these neurons. Dysfunction of channels containing the α subunit Nav1.2 may lead to alteration of brain functions, such as motor control, motivation and reward. Indeed, mutations of Nav1.2 have been associated with autism (Sanders et al., 2012; Ben-Shalom et al., 2017) and epilepsy (Kamiya et al., 2004; Lauxmann et al., 2013; Berecki et al., 2018). Thus, this channel subunit could be a potential drug target for the treatment of related brain disorders.

## Materials and Methods

### Slice Preparation

The use and care of animals in this study were approved by the Animal Advisory Committee at the State Key Laboratory of Cognitive Neuroscience and Learning, Beijing Normal University. The number of animals used in the experiments was minimized to comply the rules of ethics committee. We used TH-GFP mice (P16-25) for electrophysiological experiments. Mice were anesthetized by sodium pentobarbital (100 mg/kg, i.p.) before decapitation. The brain was dissected out and immersed in an ice-cold slicing solution (in mM: Sucrose 213, KCl 2.5, NaH_2_PO_4_ 1.25, NaHCO_3_ 26, Dextrose 10, MgSO_4_ 2, CaCl_2_ 2), which was bubbled with mixed gas (95% O_2_, 5% CO_2_). In this sucrose-based solution, brain slices (200 μm in thickness) containing the midbrain were cut on a vibratome (VT1200S, Leica). Slices were then transferred to an incubator containing the artificial cerebrospinal fluid (ACSF) (in mM: NaCl 126, KCl 2.5, NaH_2_PO_4_ 1.25, NaHCO_3_ 26, Dextrose 25, MgSO_4_ 2, CaCl_2_ 2; 315–325 mOsm, pH = 7.2–7.3) and maintained at 34°C for ∼30–60 min. After incubation, slices were kept in the same solution at the room temperature until use.

### Electrophysiological Recordings

To obtain electrophysiological recordings, we transferred slices to a recording chamber perfused with aerated ACSF at 24–26°C. The GFP-positive cells were considered as putative dopaminergic neurons. They were visualized under an infrared differential interference contrast (IR-DIC) fluorescence microscope (BX51WI, Olympus). Patch pipettes had an impedance of ∼5 MΩ for somatic recording and ∼10 MΩ for axonal bleb recording. Blebs formed at the AIS (60 μm away from the branching point of the ABD) were chosen for recording in this study. The TH-positive axons together with their blebs were identified by the expression of GFP. For current clamp recording, pipettes were filled with a K^+^-based internal solution (in mM: Kgluconate 140, KCl 3, MgCl2 2, HEPES 10, EGTA 0.2, Na2ATP 2; 295–305 mOsm, pH = 7.2). For voltage clamp recordings, pipettes were filled with a Cs^+^-based solution (in mM: CsCl 140, MgCl_2_ 2, HEPES 10, EGTA 10, Na_2_ATP 2; 295–305 mOsm, pH = 7.2). To obtain local Na^+^ currents at the soma (nucleated patch) and the axon (isolated axonal bleb), we added 4-AP (3 mM), TEA-Cl (96 mM) and CdCl_2_ (100 μM) to the ACSF but reduced the concentration of NaCl to 50 mM. Isolation of blebs from the axon was achieved as described previously (Chaudhury et al., 2013). In brief, we swept a patch pipette just below the selected GFP-positive bleb and disconnect it from the main axon. A MultiClamp 700B amplifier (Molecular Devices) was used for whole-cell recording and giant patch (somatic nucleated patch and isolated axonal bleb) recording. Voltage and current signals were acquired by pClamp10 software at a sampling rate of 50 kHz. Na^+^ current traces were averaged and then filtered with a Gaussian filter. The liquid junction potentials were not corrected for *V*m values shown in the text and figures.

The holding potential of somatic nucleated patches and isolated axonal blebs was −60 mV. For the measurement of Na^+^ channel density and the investigation of voltage-dependent channel activation, the *V*_m_ was stepped from a prepulse (−120 mV, 30 ms) to a series of testing pulses ranging from −70 to +30 mV with increment of 5 mV. The maximal peak amplitude among the evoked families of currents and the membrane area (based on the measured diameter) were used for the estimation of Na^+^ channel conductance density. The peak amplitudes at different voltage levels were normalized to the maximal amplitude and plotted as a function of the membrane potential (activation curves). We fitted the rise and decay of the evoked currents with single exponential functions to examine the kinetics of channel activation and inactivation. For channel deactivation, 0.2 ms after stepping the membrane potential from −120 to 0 mV, a series of 25-ms voltage pulses from −100 to −30 mV were applied to the patches. The deactivation time constants could be obtained from the tail currents at different voltage levels.

To examine the steady-state inactivation of Na^+^ channels, we evoked transient Na^+^ currents by stepping the *V*_m_ from a series of prepulses (from −120 to −60 mV, 35 ms) to the 20-ms test pulse at 0 mV. The evoked currents were normalized to the maximal peak amplitude to generate the inactivation curves, i.e., channel availability at different voltages. To examine the time course of channel inactivation onset, we stepped the *V*_m_ from a prepulse at −120 to −55 mV and stepped the *V*_m_ further to a test pulse of 0 mV after a delay ranging from 0 to 38 ms. Because the recordings from isolated blebs were normally stable for much longer time than that of nucleated patches, we chose to apply relatively longer time intervals in bleb recordings. Channels were supposed to inactivate at −55 mV but in a time-dependent manner. For recovery from inactivation, we applied two paired pulses from −120 to 0 mV (20 ms) with time intervals ranging from 1 to 110 ms. The first pulse in each paired steps lasted for 30 ms. Currents were normalized to the peak amplitude of the current evoked by the first pulse.

### Immunostaining

After anesthesia, animals were perfused through heart with an ice-cold fixative: 0.5∼1% paraformaldehyde (PFA) and 0.5∼1% sucrose (in 0.1 M phosphate buffer, PB). Brain tissues were dissected out and immersed in the same fixative for 2 h. Then the tissues were transferred to 30% sucrose and maintained at 4°C overnight. Tissues were cut into 30 μm-thick sections on a freezing microtome at −20°C. After wash with 0.01 M phosphate buffered saline (PBS), the sections were treated with 0.5% Triton X-100 in PB for 30 min, and then 5% bovine serum albumin and 0.5% Triton for ∼60 min at room temperature. Sections were then incubated in 0.1% Triton solution containing the primary antibodies (mouse anti-Nav1.1, 1:200, NeuroMab, 73-023; rabbit anti-Nav1.6, 1:400, Alomone Labs, ASC-009; mouse anti-Nav1.2, 1:200, NeuroMab, 73-024; goat anti-AnkG, 1:400, Santa Cruz, sc-31778; rabbit Anti-TH,1:400, Millipore, AB152; Mouse anti-TH, 1:400, Millipore, MAB318) at 4°C overnight. After complete wash with 0.01 M PBS, sections were incubated in 0.1% Triton solution containing secondary antibodies (1:1000, Invitrogen: Alexa-488 conjugated donkey anti-rabbit, A21206, Alexa-555 conjugated donkey anti-mouse, A31570, and Alexa-647 conjugated donkey anti-goat, A21447) for 2 hr at the room temperature.

We chose to image non-successive sections (about 2 mm (2 mm) for each experiment so that no cell on the surface would be over-counted. Images were taken on a laser scanning confocal microscope (A1plus, Nikon) with 10×, 20×, and 60× objectives. The acquisition parameters were carefully adjusted to make the fluorescence signals linearly displayed and fall into the maximum dynamic range of the detectors. Z-stack images were collected with a voxel interval of 1 μm.

### Statistics

All values were presented as mean ± SEM. Statistical significance of difference was examined using non-parametric Wilcoxon rank-sum test.

## Data Availability

All datasets generated for this study are included in the manuscript and/or the supplementary files.

## Ethics Statement

This study was carried out in accordance with the recommendations of the Animal Advisory Committee at the State Key Laboratory of Cognitive Neuroscience and Learning, Beijing Normal University. The protocol was also approved by this committee.

## Author Contributions

YS initiated and designed the experiments. JY performed all the patch-clamp recordings and analyzed the data. YX helped in dual soma-axon recordings. LL and ML conducted the immunostaining experiments. QH helped in data analysis. JY and YS wrote the manuscript.

## Conflict of Interest Statement

The authors declare that the research was conducted in the absence of any commercial or financial relationships that could be construed as a potential conflict of interest.

## References

[B1] Ben-Shalom R. Keeshen C. M. Berrios K. N. An J. Y. Sanders S. J. Bender K. J. (2017). Opposing effects on NaV1.2 function underlie differences between SCN2A variants observed in individuals with autism spectrum disorder or infantile seizures. *Biol. Psychiatry* 82 224–232. 10.1016/j.biopsych.2017.01.009 28256214 PMC5796785

[B2] Berecki G. Howell K. B. Deerasooriya Y. H. Cilio M. R. Oliva M. K. Kaplan D. (2018). Dynamic action potential clamp predicts functional separation in mild familial and severe de novo forms of SCN2A epilepsy. *Proc. Natl. Acad. Sci. U.S.A.* 115 E5516–E5525. 10.1073/pnas.1800077115 29844171 PMC6004444

[B3] Bermudez M. A. Schultz W. (2010). Reward magnitude coding in primate amygdala neurons. *J. Neurophysiol.* 104 3424–3432. 10.1152/jn.00540.2010 20861431 PMC3007636

[B4] Cantrell A. R. Catterall W. A. (2001). Neuromodulation of Na+ channels: an unexpected form of cellular plasticity. *Nat. Rev. Neurosci.* 2 397–407. 10.1038/35077553 11389473

[B5] Cao J. L. Covington H. E. III Friedman A. K. Wilkinson M. B. Walsh J. J. Cooper D. C. (2010). Mesolimbic dopamine neurons in the brain reward circuit mediate susceptibility to social defeat and antidepressant action. *J. Neurosci.* 30 16453–16458. 10.1523/JNEUROSCI.3177-10.2010 21147984 PMC3061337

[B6] Carr D. B. Day M. Cantrell A. R. Held J. Scheuer T. Catterall W. A. (2003). Transmitter modulation of slow, activity-dependent alterations in sodium channel availability endows neurons with a novel form of cellular plasticity. *Neuron* 39 793–806. 10.1016/s0896-6273(03)00531-2 12948446

[B7] Chan A. W. Owens S. Tung C. Stanley E. F. (2007). Resistance of presynaptic CaV2.2 channels to voltage-dependent inactivation: dynamic palmitoylation and voltage sensitivity. *Cell Calcium* 42 419–425. 10.1016/j.ceca.2007.04.009 17602741

[B8] Chan C. S. Gertler T. S. Surmeier D. J. (2009). Calcium homeostasis, selective vulnerability and Parkinson’s disease. *Trends Neurosci.* 32 249–256. 10.1016/j.tins.2009.01.006 19307031 PMC4831702

[B9] Chaudhury D. Walsh J. J. Friedman A. K. Juarez B. Ku S. M. Koo J. W. (2013). Rapid regulation of depression-related behaviours by control of midbrain dopamine neurons. *Nature* 493 532–536. 10.1038/nature11713 23235832 PMC3554860

[B10] Chen G. Henter I. D. Manji H. K. (2009). A role for PKC in mediating stress-induced prefrontal cortical structural plasticity and cognitive function. *Proc. Natl. Acad. Sci. U.S.A.* 106 17613–17614. 10.1073/pnas.0909771106 19828441 PMC2764949

[B11] Chen Y. Yu F. H. Sharp E. M. Beacham D. Scheuer T. Catterall W. A. (2008). Functional properties and differential neuromodulation of Na(v)1.6 channels. *Mol. Cell. Neurosci.* 38 607–615. 10.1016/j.mcn.2008.05.009 18599309 PMC3433175

[B12] Chen Y. Yu F. H. Surmeier D. J. Scheuer T. Catterall W. A. (2006). Neuromodulation of Na+ channel slow inactivation via cAMP-dependent protein kinase and protein kinase C. *Neuron* 49 409–420. 10.1016/j.neuron.2006.01.009 16446144

[B13] Clark B. A. Monsivais P. Branco T. London M. Hausser M. H. (2005). The site of action potential initiation in cerebellar Purkinje neurons. *Nat. Neurosci.* 8 137–139. 10.1038/nn1390 15665877

[B14] Cragg S. J. Greenfield S. A. (1997). Differential autoreceptor control of somatodendritic and axon terminal dopamine release in substantia nigra, ventral tegmental area, and striatum. *J. Neurosci.* 17 5738–5746. 10.1523/JNEUROSCI.17-15-05738.1997 9221772 PMC6573186

[B15] Dauer W. Przedborski S. (2003). Parkinson’s disease: mechanisms and models. *Neuron* 39 889–909. 10.1016/S0896-6273(03)00568-3 12971891

[B16] Ding S. Wei W. Zhou F. M. (2011). Molecular and functional differences in voltage-activated sodium currents between GABA projection neurons and dopamine neurons in the substantia nigra. *J. Neurophysiol.* 106 3019–3034. 10.1152/jn.00305.2011 21880943 PMC3234097

[B17] Fields H. L. (2007). Understanding how opioids contribute to reward and analgesia. *Reg. Anesth. Pain. Med.* 32 242–246. 10.1016/j.rapm.2007.01.001 17543821

[B18] Geffen L. B. Jessell T. M. Cuello A. C. Iversen L. L. (1976). Release of dopamine from dendrites in rat substantia nigra. *Nature* 260 258–260. 10.1038/260258a0 1256567

[B19] Gentet L. J. Williams S. R. (2007). Dopamine gates action potential backpropagation in midbrain dopaminergic neurons. *J. Neurosci.* 27 1892–1901. 10.1523/JNEUROSCI.5234-06.2007 17314285 PMC6673536

[B20] Gonzalez-Cabrera C. Meza R. Ulloa L. Merino-Sepulveda P. Luco V. Sanhueza A. (2017). Characterization of the axon initial segment of mice substantia nigra dopaminergic neurons. *J. Comp. Neurol.* 525 3529–3542. 10.1002/cne.24288 28734032

[B21] Grace A. A. Bunney B. S. (1984). The control of firing pattern in nigral dopamine neurons: burst firing. *J. Neurosci.* 4 2877–2890. 10.1523/JNEUROSCI.04-11-02877.1984 6150071 PMC6564720

[B22] Grace A. A. Floresco S. B. Goto Y. Lodge D. J. (2007). Regulation of firing of dopaminergic neurons and control of goal-directed behaviors. *Trends. Neurosci.* 30 220–227. 10.1016/j.tins.2007.03.003 17400299

[B23] Gunaydin L. A. Grosenick L. Finkelstein J. C. Kauvar I. V. Fenno L. E. Adhikari A. (2014). Natural neural projection dynamics underlying social behavior. *Cell* 157 1535–1551. 10.1016/j.cell.2014.05.017 24949967 PMC4123133

[B24] Hausser M. Stuart G. Racca C. Sakmann B. (1995). Axonal initiation and active dendritic propagation of action potentials in substantia nigra neurons. *Neuron* 15 637–647. 10.1016/0896-6273(95)90152-3 7546743

[B25] Hounsgaard J. Nedergaard S. Greenfield S. A. (1992). Electrophysiological localization of distinct calcium potentials at selective somatodendritic sites in the substantia nigra. *Neuroscience* 50 513–518. 10.1016/0306-4522(92)90443-6 1331866

[B26] Hu H. Gan J. Jonas P. (2014). Interneurons. Fast-spiking, parvalbumin(+) GABAergic interneurons: from cellular design to microcircuit function. *Science* 345:1255263. 10.1126/science.1255263 25082707

[B27] Hu W. Tian C. Li T. Yang M. Hou H. Shu Y. (2009). Distinct contributions of Na(v)1.6 and Na(v)1.2 in action potential initiation and backpropagation. *Nat. Neurosci.* 12 996–1002. 10.1038/nn.2359 19633666

[B28] Johnson S. W. Seutin V. North R. A. (1992). Burst firing in dopamine neurons induced by N-methyl-D-aspartate: role of electrogenic sodium pump. *Science* 258 665–667. 10.1126/science.1329209 1329209

[B29] Kamiya K. Kaneda M. Sugawara T. Mazaki E. Okamura N. Montal M. (2004). A nonsense mutation of the sodium channel gene SCN2A in a patient with intractable epilepsy and mental decline. *J. Neurosci.* 24 2690–2698. 10.1523/JNEUROSCI.3089-03.2004 15028761 PMC6729532

[B30] Khaliq Z. M. Bean B. P. (2010). Pacemaking in dopaminergic ventral tegmental area neurons: depolarizing drive from background and voltage-dependent sodium conductances. *J. Neurosci.* 30 7401–7413. 10.1523/JNEUROSCI.0143-10.2010 20505107 PMC2892804

[B31] Kole M. H. Ilschner S. U. Kampa B. M. Williams S. R. Ruben P. C. Stuart G. J. (2008). Action potential generation requires a high sodium channel density in the axon initial segment. *Nat. Neurosci.* 11 178–186. 10.1038/nn2040 18204443

[B32] Koob G. F. (1998). Circuits, drugs, and drug addiction. *Adv. Pharmacol.* 42 978–982. 10.1016/s1054-3589(08)60910-2 9328061

[B33] Korotkova T. M. Sergeeva O. A. Eriksson K. S. Haas H. L. Brown R. E. (2003). Excitation of ventral tegmental area dopaminergic and nondopaminergic neurons by orexins/hypocretins. *J. Neurosci.* 23 7–11. 10.1523/JNEUROSCI.23-01-00007.2003 12514194 PMC6742159

[B34] Kuwahara T. Koyama A. Gengyo-Ando K. Masuda M. Kowa H. Tsunoda M. (2006). Familial Parkinson mutant alpha-synuclein causes dopamine neuron dysfunction in transgenic *Caenorhabditis elegans*. *J. Biol. Chem.* 281 334–340. 10.1074/jbc.M504860200 16260788

[B35] Lauxmann S. Boutry-Kryza N. Rivier C. Mueller S. Hedrich U. B. Maljevic S. (2013). An SCN2A mutation in a family with infantile seizures from Madagascar reveals an increased subthreshold Na(+) current. *Epilepsia* 54 e117–e121. 10.1111/epi.12241 23758435

[B36] Lewis D. A. Lieberman J. A. (2000). Catching up on schizophrenia: natural history and neurobiology. *Neuron* 28 325–334. 10.1016/S0896-6273(00)00111-2 11144342

[B37] Li T. Tian C. Scalmani P. Frassoni C. Mantegazza M. Wang Y. (2014). Action potential initiation in neocortical inhibitory interneurons. *PLoS Biol.* 12:e1001944. 10.1371/journal.pbio.1001944 25203314 PMC4159120

[B38] Margolis E. B. Lock H. Hjelmstad G. O. Fields H. L. (2006). The ventral tegmental area revisited: is there an electrophysiological marker for dopaminergic neurons? *J. Physiol.* 577 907–924. 10.1113/jphysiol.2006.117069 16959856 PMC1890372

[B39] Maurice N. Tkatch T. Meisler M. Sprunger L. K. Surmeier D. J. (2001). D1/D5 dopamine receptor activation differentially modulates rapidly inactivating and persistent sodium currents in prefrontal cortex pyramidal neurons. *J. Neurosci.* 21 2268–2277. 10.1523/JNEUROSCI.21-07-02268.2001 11264302 PMC6762404

[B40] McCormick D. A. Shu Y. Yu Y. (2007). Neurophysiology: Hodgkin and Huxley model–still standing? *Nature* 445 E1–E2. 10.1038/nature05523 17203021

[B41] Moubarak E. Engel D. Dufour M. A. Tapia M. Tell F. Goaillard J. M. (2019). Robustness to axon initial segment variation is explained by somatodendritic excitability in rat substantia nigra dopaminergic neurons. *J. Neurosci.* 39 5044–5063. 10.1523/JNEUROSCI.2781-18.2019 31028116 PMC6595954

[B42] Neuhoff H. Neu A. Liss B. Roeper J. (2002). I(h) channels contribute to the different functional properties of identified dopaminergic subpopulations in the midbrain. *J. Neurosci.* 22 1290–1302. 10.1523/JNEUROSCI.22-04-01290.2002 11850457 PMC6757558

[B43] Nieoullon A. (2002). Dopamine and the regulation of cognition and attention. *Prog. Neurobiol.* 67 53–83. 10.1016/s0301-0082(02)00011-4 12126656

[B44] Rice M. E. Patel J. C. (2015). Somatodendritic dopamine release: recent mechanistic insights. *Philos. Trans. R. Soc. Lond. B Biol. Sci.* 370:20140185. 10.1098/rstb.2014.0185 26009764 PMC4455754

[B45] Rush A. M. Dib-Hajj S. D. Waxman S. G. (2005). Electrophysiological properties of two axonal sodium channels, Nav1.2 and Nav1.6, expressed in mouse spinal sensory neurones. *J. Physiol.* 564 803–815. 10.1113/jphysiol.2005.083089 15760941 PMC1464456

[B46] Sanders S. J. Murtha M. T. Gupta A. R. Murdoch J. D. Raubeson M. J. Willsey A. J. (2012). De novo mutations revealed by whole-exome sequencing are strongly associated with autism. *Nature* 485 237–241. 10.1038/nature10945 22495306 PMC3667984

[B47] Seutin V. Engel D. (2010). Differences in Na+ conductance density and Na+ channel functional properties between dopamine and GABA neurons of the rat substantia nigra. *J. Neurophysiol.* 103 3099–3114. 10.1152/jn.00513.2009 20357070

[B48] Shah B. S. Stevens E. B. Pinnock R. D. Dixon A. K. Lee K. (2001). Developmental expression of the novel voltage-gated sodium channel auxiliary subunit beta3, in rat CNS. *J. Physiol.* 534 763–776. 10.1111/j.1469-7793.2001.t01-1-00763.x 11483707 PMC2278751

[B49] Stuart G. Spruston N. Sakmann B. Hausser M. (1997). Action potential initiation and backpropagation in neurons of the mammalian CNS. *Trends. Neurosci.* 20 125–131. 10.1016/S0166-2236(96)10075-8 9061867

[B50] Sulzer D. Cragg S. J. Rice M. E. (2016). Striatal dopamine neurotransmission: regulation of release and uptake. *Basal Ganglia* 6 123–148. 10.1016/j.baga.2016.02.001 27141430 PMC4850498

[B51] Tobler P. N. O’Doherty J. P. Dolan R. J. Schultz W. (2007). Reward value coding distinct from risk attitude-related uncertainty coding in human reward systems. *J. Neurophysiol.* 97 1621–1632. 10.1152/jn.00745.2006 17122317 PMC2637604

[B52] Tsai H. C. Zhang F. Adamantidis A. Stuber G. D. Bonci A. de Lecea L. (2009). Phasic firing in dopaminergic neurons is sufficient for behavioral conditioning. *Science* 324 1080–1084. 10.1126/science.1168878 19389999 PMC5262197

[B53] Tye K. M. Mirzabekov J. J. Warden M. R. Ferenczi E. A. Tsai H. C. Finkelstein J. (2013). Dopamine neurons modulate neural encoding and expression of depression-related behaviour. *Nature* 493 537–541. 10.1038/nature11740 23235822 PMC4160519

[B54] West J. W. Numann R. Murphy B. J. Scheuer T. Catterall W. A. (1991). A phosphorylation site in the Na+ channel required for modulation by protein kinase C. *Science* 254 866–868. 10.1126/science.1658937 1658937

[B55] Wise R. A. (2004). Rewards wanted: molecular mechanisms of motivation. *Discov. Med.* 4 180–186. 20704982

[B56] Ye M. Y. Yang J. Tian C. P. Zhu Q. Y. Yin L. P. Jiang S. (2018). Differential roles of Na(V)1.2 and Na(V)1.6 in regulating neuronal excitability at febrile temperature and distinct contributions to febrile seizures. *Sci. Rep.* 8:753. 10.1038/s41598-017-17344-8 29335582 PMC5768682

